# Application of solid-state fermentation by microbial biotechnology for bioprocessing of agro-industrial wastes from 1970 to 2020: A review and bibliometric analysis

**DOI:** 10.1016/j.heliyon.2022.e09173

**Published:** 2022-03-24

**Authors:** Levi Yafetto

**Affiliations:** Department of Molecular Biology and Biotechnology, School of Biological Sciences, College of Agriculture and Natural Sciences, University of Cape Coast, Cape Coast, Ghana

**Keywords:** Agro-industrial residues, Bibliometrics, Bioprocessing, Microbial biotechnology, Solid-state fermentation, Valorization

## Abstract

This paper reviews the pertinent literature from 1970 to 2020 and presents a bibliometric analysis of research trends in the application of solid-state fermentation in the bioprocessing of agro-industrial wastes. A total 5630 publications of studies on solid-state fermentation that comprised of 5208 articles (92.50%), 340 book chapters (6.04%), 39 preprints (0.69%), 32 proceedings (0.56%), 8 edited books (0.14%) and 3 monographs (0.05%) were retrieved from Dimensions database. A review of the literature indicated that (i) fermentation of solid substrates is variously defined in the literature over the past 50 years, where “solid-state fermentation” is the most dominant research term used, and (ii) key products derived from the valorization of agro-industrial wastes through solid-state fermentation include, among others, enzymes, antioxidants, animal feed, biofuel, organic acids, biosurfactants, etc. Bibliometric analyses with VOSviewer revealed an astronomic increase in publications between 2000 and 2020, and further elucidated the most frequently explored core research topics, the most highly cited publications and authors, and countries/regions with the highest number of citations. The most cited publication between 2010 and 2020 had 382 citations compared to 725 citations for the most cited publication from 1970 to 2020. Ashok Pandey from India was the most published and cited author with 123 publications and 8,613 citations respectively; whereas *Bioresource Technology* was the most published and cited journal with 233 publications and 12,394 citations. Countries with the most publications and citations are Brazil, France, India, and Mexico. These findings suggest that research in the application of solid-state fermentation for bioprocessing of agro-industrial wastes has gained prominence over the past 50 years. Future perspectives and implications are discussed.

## 1 Introduction

Fermentation has been practiced over many centuries to produce bread, beer, cheese and wine. Through human activities and experience, cereals, root tubers, and fruits and vegetables have been used to produce fermented solid foods and alcoholic and non-alcoholic beverages. Therefore, over many decades, the application of fermentation has led to the production of a variety of fermented foods that are popular among indigenous peoples and cultures around the world. For example, sake, miso, soy sauce, tempeh, tapai, and koji, are popular fermented foods produced in Asia; sauerkraut, tabasco sauce, chichi and champú are popular fermented cuisines in Europe and America; in Africa, particularly Ghana, fermented foods like *gari* (fermented cassava grits), *kenkey* (fermented, boiled corn dough), *kokonte* (fermented cassava chips), *kooko* (fermented corn porridge), *fura* (fermented millet dough), *wagashi* (a traditional West African cottage cheese), and *pito* and *brukutu* (fermented African beer from sorghum) are some of the popular fermented foods consumed in most households (Egwim et al., 2013; Manan and Webb, 2017). These fermented foods, mostly obtained from solid agricultural staples, are prized for their cultural, culinary, economic, and nutritional values. They remain a central part of most cuisines in particularly sub-Saharan Africa (Hesseltine, 1979; Lyons, 2007).

Solid-state fermentation is a microbial fermentation process through which selected microorganisms (bacteria, fungi and yeasts) are cultivated on a moist, solid, non-soluble organic material that acts as a support and nutrient source for the growth of the microorganisms, in the absence or near absence of free-flowing water (Pandey et al., 2000a; Manan and Webb, 2017). It is considered an important, viable food processing approach for bioconversion of organic agro-industrial wastes (Manan and Webb, 2017). Globally, the food, pharmaceutical, energy, and chemical industries are the main beneficiaries of the application of solid-state fermentation, because, through microbial biotechnology, it is conveniently used in the production of fermented foods and other useful industrial products (Couto and Sanromán, 2006; Ghosh, 2016). Through research and industrialization in the mid-twentieth century, the food industry witnessed a rapid increase in the utilization of solid-state fermentation that transformed the industrial fortunes of most countries in the world. To this end, other industries have actively utilized agro-industrial wastes to produce nutrient-rich fermented animal feed, organic acids, antibiotics, bioethanol, mushrooms, antioxidants, single-cell proteins, enzymes, secondary metabolites, biofuels and, more recently, biosurfactants, which are used in the bioremediation of environmental pollution as a result of indiscriminate disposal of agro-industrial wastes (Pandey et al., 2000a, b, c, d; Soccol and Vandenberghe 2003; Khopade et al., 2012; Lizardi-Jimenez and Hernandez-Martınez, 2017; Yafetto et al., 2019). The application of solid-state fermentation is feasible because of its usefulness to convert different agro-lignocellulosic substrates – straws, husks and brans of cereals, bagasse, molasses, oil cakes, the peels and pulps of tubers, fruits and vegetables, paper pulp, etc., – into some of the industrial products aforementioned (Obi et al., 2016). Most of the microorganisms used in fermentation – particularly filamentous fungi and yeasts – are generally regarded as safe, i.e., their involvement in solid-state fermentation renders the final products free from toxins, thereby making the products safe for consumption by animals and humans (Suman et al., 2015; Upadhyaya et al., 2016; Yafetto et al., 2019).

**Figure 1:**
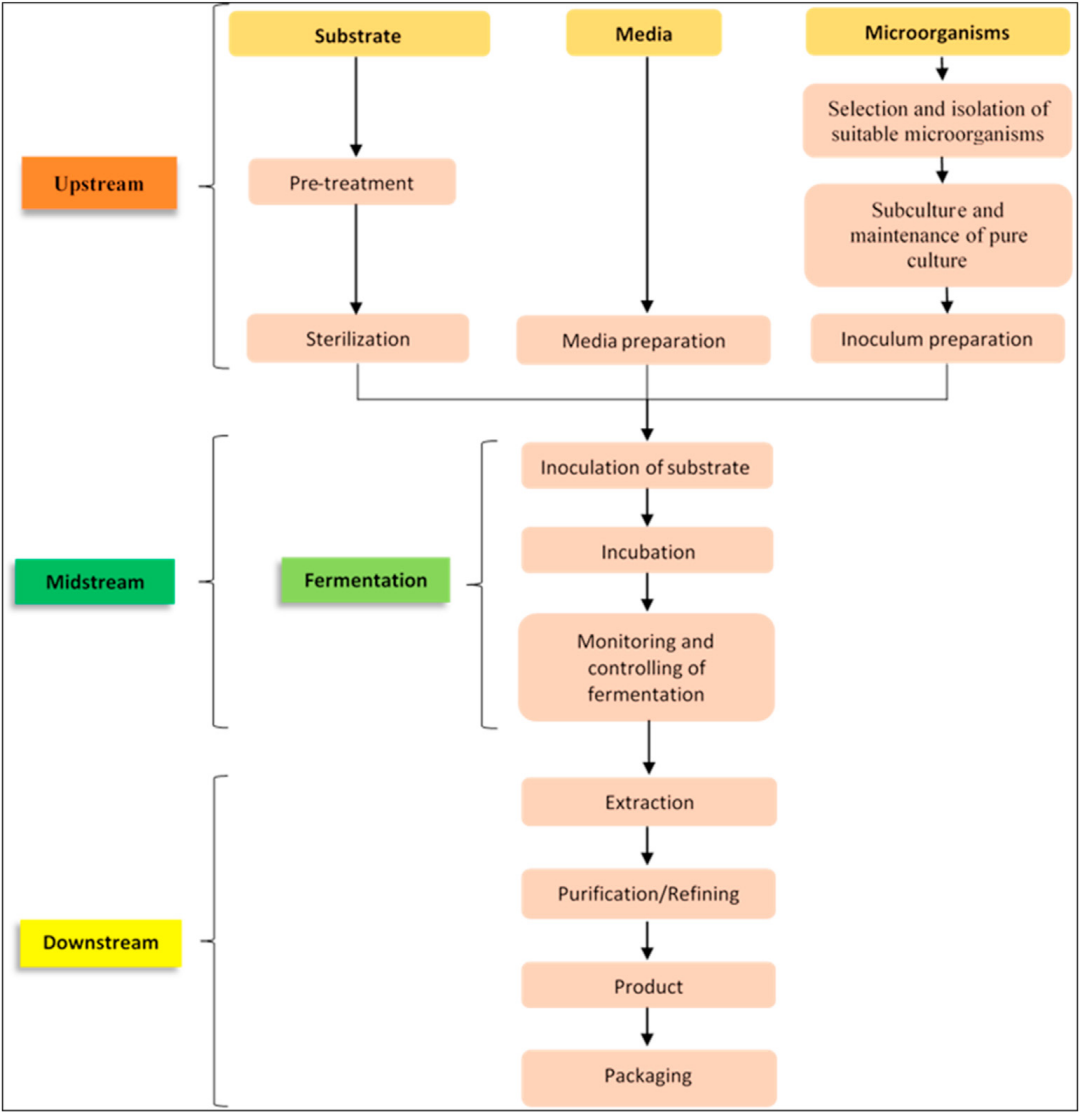
A chart flow of steps involved in solid-state fermentation.

Solid-state fermentation involves a series of steps that are characterized into upstream, midstream, and downstream processes (Mitchell et al., 2000; Ashok et al., 2017). The upstream process involves the preparation of substrates and growth media, and the isolation of microorganisms used for the fermentation, followed by the midstream process during which the substrate is inoculated and fermented, and then the downstream process where the final products obtained are processed for packaging (Figure 1). Although the steps involved in solid-state fermentation are widely used in industry, there are slight differences in the approaches to achieving the final desired product. Nambi et al. (2017) and Yafetto et al. (2019) recently used slightly modified approaches for solid-state fermentation studies that aimed to enrich the protein contents of grains and cassava peels. These differences in approach notwithstanding, solid-state fermentation is technologically feasible across the board, but at different stages of development, both at the laboratory level and on pilot scales (Lin et al., 2020; Ŝelo et al., 2021; Wang et al., 2022). To this end, several studies have been conducted in countries whose economies are predominantly dependent on agriculture (Ravindran et al., 2018) to develop state-of-the-art technologies for waste valorization as alternatives to conventional waste management strategies (Wang et al., 2022). To sustain the waste valorization, assessment of environmental, social and economic impacts of the emerging valorization technologies through life cycle assessment (LCA) and techno-economic analysis (TEA) is critical (Al-Wahaibi et al., 2020; Osman et al., 2021; Wang et al., 2022). Osman et al. (2021) and Wang et al. (2022) have reviewed comprehensively the major waste valorization approaches of selected waste streams and products obtained from them.

Solid-state fermentation has, over the decades, attained global recognition because of its potential to contribute significantly to solving some of the world's persistent problems, including malnutrition in humans and livestock, environmental pollution, climate change, hunger, and improving global food security (Ezekiel and Aworh, 2013; Zepf and Jin, 2013; FAO, 2016; Meybeck et al., 2018; Parmar et al., 2019). To this end, many studies on solid-state fermentation have culminated in published findings as demonstrated by the primary literature. It is, however, surprising that there is no bibliometric analysis of the literature on solid-state fermentation, as a field, based on findings from data mined in the various research databases. Rather, there exist some bibliometric studies on science or social science as general fields, with few discipline-specific studies that have exclusively examined and compared research productivity and impact among scholars and nations (Zhou et al., 2009; Bajwa and Yaldram, 2013; Xie and Willett, 2013; Liu et al., 2015a, b, Lei and Liu, 2019). These studies, however, lack some details of the most relevant issues related to the fields such as (i) the most frequently explored research topics, (ii) publications with the most citations, and (iii) researchers with the most contributions to the body of knowledge in the field, among others. Given that there has not been such discipline-specific bibliometric research on solid-state fermentation, this paper, therefore, sought to (i) review the literature on solid-state fermentation between 2000 and 2020, and, (ii) conduct a bibliometric analysis of the literature between 1970 and 2020, specifically, to answer the following questions:

(i) what are the major types of publications?
(ii) what are the most frequently explored topics?
(iii) what are the most highly cited publications and authors?
(iv) what are the most highly cited journals? and
(v) which country/region has the most cited publications?

## 2 Literature search and analysis

A comprehensive search of the primary scientific literature was conducted on 17^th^ March 2021 in Dimensions (https://www.dimensions.ai), an online linked research knowledge system using the search term “solid-state fermentation”. The search was further modified to include other terms like “animal feed”, “biofertilizers”, “enzymes”, “antioxidants”, “biofuel”, etc., to extract specific scientific literature on the most frequently explored topics in solid-state fermentation based on the end-products. The following filters were manually activated for the search: (i) Relevance, and (ii) Title and Abstract. Dimensions automatically selected the following filters for the search: (i) Year of Publication, which ranged between 1970 and 2021 (ii) Researchers, (iii) Research Categories, (iv) Research Type, (v) Source Titles, and (vi) Journal List. Articles published between 2000 and 2020 were selected for a review of the literature. To determine the most highly cited publications and authors, and the research trends in the field between 1970 and 2020, automatically-generated statistical details of all cited publications (journal articles, books, book chapters, preprints, proceedings and monographs) were retrieved in the “Analytical Views” section of Dimensions and processed with Microsoft Excel. All publications on solid-state fermentation between 1970 and 2020 were manually selected and exported to the Export Center of Dimensions. Subsequently, the Excel files were exported to VOSviewer (Version 1.6.17) for bibliometric analyses and network visualizations of the most cited author, the most cited journal, and the country with most cited publications as described by van Eck and Waltman (2010).

## 3 Solid-state fermentation

Solid-state fermentation has been variously defined in the past two decades by researchers without much deviation from the basic fundamental principles that outline the fermentation process (Table 1; Manan and Webb, 2017). Additionally, other different terms have been used throughout literature, over the years, to variously refer to this fermentation process. These terms include the following: (i) *solid substrate fermentation*, (ii) *solid state bioprocessing*, (iii) *solid substrate cultivation*, (iv) *solid state digestion*, (v) *solid state cultivation*, (vi) *solid-phase fermentation*, (vii) *solid state culture*, (viii) *surface cultivation*, and (iv) *surface culture* (Manan and Webb, 2017; Mitchell et al., 2000). According to Manan (2014), a search for the use of the aforementioned terms in research publication databases such as Scopus, Web of Knowledge (Web of Science and All Database), and ScienceDirect between the period of 1971–2014 revealed that “solid-state fermentation” is the most commonly used term to describe the fermentation process, followed by “solid substrate fermentation”. A similar search was conducted in the Dimensions database to analyze the literature from 1970 to 2020 and retrieved a total number of 7584 publications that involved the use of all these terms (Table 2). Interestingly, as reported by Manan (2014), search results presented in this study showed “solid-state fermentation” as the most commonly used term in 5630 publications (74.23%) (Table 2). Surprisingly, whereas “solid substrate fermentation” was the second most commonly used term reported by Manan (2014) and Manan and Webb (2017), this paper revealed that “surface culture” is now the second most commonly used term (627 publications; 8.26%), followed by “solid state culture” (441 publications; 5.81%), and “solid substrate fermentation” (362 publications: 4.77% in that order (Table 2). The data suggest further that, historically, “solid-state fermentation” has gained prominence consistently over the other terms, as a result of which it is the most commonly used term among researchers. For example, it was revealed that as of 17^th^ March 2021 when the search was conducted for this study, the terms“solid-phase fermentation” and “solid state bioprocessing” had no mention in the literature

**Table 1:** Definitions of solid-state fermentation.

Definition	Reference
A microbial process occurring mostly on the surface of solid materials that have the property to absorb or contain water, with or without soluble nutrients.	Viniegraz-Gonzàlez (1997)
Cultivation of microorganisms on moist solid supports, either on inert carriers or on insoluble substrates that can also be used as carbon and energy source.	Pandey et al. (2000a)
Any process in which substrates in a solid particulate state are utilized.	Mitchell et al. (2000)
The growth of microorganisms on a moistened solid substrate, in which enough moisture is present to maintain microbial growth and metabolism, but where there is no free-moving water and air is the continuous phase.	Rahardjo et al*.* (2006)
The growth of microorganisms on solid or semisolid substrates or support.	Rosales et al. (2007)
A process that involves the growth of microorganisms on moist particles of solid materials in beds in which the spaces between the particles are filled with a continuous gas phase	Mitchell et al. (2011)
A three-phase, heterogeneous process, comprising solid, liquid, and gaseous phases, which offers potential benefits for the microbial cultivation for bioprocess and products development	Thomas et al. (2013)

**Table 2:** Different terms used to describe the fermentation of solid substrates.

S/N	Term	Number of publications	Per cent in total publication
1	Solid-state fermentation	5630	74.23
2	Surface culture	627	8.26
3	Solid state culture	441	5.81
4	Solid substrate fermentation	362	4.80
5	Solid state cultivation	298	3.92
6	Surface cultivation	78	1.04
7	Solid-phase fermentation	61	0.80
8	Solid state bioprocessing	41	0.54
9	Solid substrate cultivation	36	0.47
10	Solid state digestion	10	0.13
	**Total**	**7584**	**100**

on solid-state fermentation for 2021; “solid substrate cultivation” had no mention since 2018; and “solid-state digestion” had no mention between 2015 and 2019, but was mentioned in 2020, with no mention again in 2021. It is not surprising, therefore, that the number of publications recorded for these terms in the search was low (Table 2). Notwithstanding the various definitions and the use of the different terms in literature, Manan and Webb (2017) assert that solid-state fermentation is a microbial process that occurs in the absence or near absence of free water, closely mimicking the natural environment, to which the selected microorganisms, especially fungi, are naturally adapted.

## 4 Microorganisms used in solid-state fermentation

Microorganisms notably used in solid-state fermentation are mostly filamentous fungi of the genera *Aspergillus*, *Fusarium*, *Penicillium*, *Rhizopus*, and *Trichoderma*. Yeasts (*Saccharomyces cerevisiae, Saccharomyces boulardii, Candida* sp) and actinobacteria species (*Streptomyces thermonitrificans*, *Streptomyces chattanoogensis*) are also employed in solid-state fermentation (Orozco et al., 2008; Hu et al., 2012; Munishamanna et al., 2017). Bacteria, particularly *Bacillus megaterium*, *Bacillus mycoides,* and *Lactobacillus* spp such as *L. acidophilus, L. bulgaricus*, *L. plantarum, L. rhamnosus, L. delbrueckii,* and *L. coryniformis*, are equally used in solid-state fermentation (Oboh, 2006; Hongzhang et al., 2011; Hsu et al., 2013; Andriani et al., 2015; Saanu and Oladiti, 2018)*.* Filamentous fungi and yeasts digest solid organic substrates in an environment with low moisture content making them ideal for use in solid-state fermentation (Yazid et al., 2017). Interestingly, *Streptomyces* spp., which are Gram-positive mycelial bacteria, are used in solid-state fermentation because they can efficiently colonize solid organic materials, produce a plethora of degradative enzymes, and tolerate harsh environmental conditions (Orozco et al., 2008).

The choice of microorganisms for an effective fermentation process is dependent on the microorganism's growth behaviour, specific product yield, ability to breakdown a particular substrate, good tolerance to temperature and pH, amenability to genetic manipulation, and the safety of the fermented product for human and animal consumption (Suman et al., 2015; Upadhyaya et al., 2016). Research on solid-state fermentation has shown that microorganisms can be employed singly as mono-cultures, co-cultures (a combination of two or more known pure cultures), or a consortium of mixed cultures (Sadh et al., 2018). Therefore, filamentous fungi, yeasts and bacteria can be used individually in mono-cultures, or in the following permutations of co-cultures (i) filamentous fungi and bacteria, (ii) filamentous fungi and yeast, or (iii) yeast and bacteria to affect fermentation of solid substrates (Manan and Webb, 2017). Adu et al. (2018), Yafetto et al. (2019), and Yafetto et al. (2020) demonstrated that protein contents of cassava and yam substrates can be improved with mono- and co-cultures of *Aspergillus niger* and *Trichoderma viride*. Similarly, Aruna et al. (2017) employed a mono-culture of the yeast, *Saccharomyces cerevisiae*, to enhance the protein content of yam peels. In another study, Oboh (2006) also used a co-culture of *S*. *cerevisiae* (yeast) and *Lactobacillus* sp. (bacterium) to enrich the nutrient content of cassava peels. Other similar studies have been conducted and cited in the literature (Villas-Boas et al., 2003; Yalemtesfa et al., 2010; Siqueira et al., 2011; Darwish et al., 2012; Omwango et al., 2013; Maiti et al., 2016; Oliveira et al., 2017; Olorunnisola et al., 2017; Aruna et al., 2018). These studies affirm that different microorganisms can be used to valorize different substrates for the production of specific products (Tables 4, 5, and 6).

## 5 Utilization of agro-industrial wastes through solid-state fermentation

Agriculture is considered globally as a major lifeline in the economy of both developed and developing countries. Through agriculture, fresh foods are produced for human consumption and to provide raw materials for the food processing industries. However, not all agricultural produce meant for food and the industries are utilized as such. Leftovers that are generated from agricultural and industrial activities mostly go waste, including those that are produced directly on the field during harvesting, and are, therefore, regarded as agro-industrial residues (Sadh et al., 2018). Obi et al. (2016) reported that about 20% of the maize crop is utilized as food, while the remaining 80% is discarded as waste; they further classified agricultural wastes into those obtained from food processing industries, crop residues, and fruits and vegetables. According to Bharathiraja et al. (2017), an estimated 5 billion metric tons of agricultural wastes are generated globally per annum from groundnut cake, rice bran, rice straw, sugarcane bagasse, fruits and vegetable wastes, wheat bran, cotton leaf scraps, etc. Ravindran et al. (2018) in a review suggested that, globally, one-third of food meant for human consumption (i.e. about 1.3 billion tonnes) goes waste. Of this waste, fruits and vegetables, and roots and tubers contribute the highest quantity between 520–650 million tonnes. Most of these wastes are casually utilized on farms as beddings or livestock feed for farm animals or carted away to be used by other peasant farmers or animal breeders (Adu et al., 2018; Ravindran et al., 2018). The remaining agro-industrial wastes are usually dumped, burnt or buried either at farm sites or at the backyards of food-processing industries, polluting the environment in the process (Iyayi and Losel 2001; Adu et al., 2018; Sadh et al., 2018). These practices of managing agro-industrial wastes – dumping, burning and burying – are commonplace because they are employed to get rid of field wastes to prepare farmlands for the next planting season, and to cut down the high cost of waste management (Sharma et al., 2020). But because (i) dumping and burying of agro-industrial wastes lead to the provision of necessary conditions for the growth of disease-causing microorganisms and disease-spreading flies, and (ii) burning releases toxic gases into the atmosphere, there is the need to find and employ an appropriate environmentally friendly, low-cost, and economically viable approach to managing agro-industrial wastes that are beneficial to humans, animals and the environment (Spalvins et al., 2018). One solution to solving this challenge, although it has its limitations, is the conventional option of directly using agricultural wastes as beddings and fodder for livestock and fuel in small-scale cottage industries (Obi et al., 2016; Ravindran et al., 2018). This approach may result in undernourishment and malnutrition since most agro-industrial wastes generally have high fibre with low nutritional contents of proteins, carbohydrates, and fat so they are deemed to be of poor feed quality (Obi et al., 2016). The other solution that is profoundly unique is the bioconversion and valorization of agro-industrial wastes with microorganisms through solid-state fermentation. The agro-industrial wastes, including forestry residues such as sawdust, present themselves as suitable candidates for utilization in solid-state fermentation because they are plentiful, cheap, readily available, easy to collect from farm sites or industry, and easy to prepare for their intended use. Besides, the choice of agro-industrial wastes for use in solid-state fermentation is also dependent on their composition, i.e. sugars, starch, proteins, cellulosic (35–50%), hemicellulosic (25–30%) or lignocellulosic (15–25%) contents (Table 3; Manpreet et al., 2005; Behera and Ray, 2016; Manan and Webb, 2017; Soccol et al., 2017). Soccol et al. (2017) suggest that the distinction in substrate composition is critical to the success of the fermentation process because specific microorganisms make use of different substrates. For effective utilization by microorganisms, substrates are usually mechanically broken down, through secretion and activities of enzymes, into smaller particles to facilitate mycelial penetration and colonization. The substrates serve as carbon and energy sources for microorganisms to utilize to synthesize cellular components (Zepf and Jin, 2013). According to Couto (2008), the substrate does not only serve as a source of nutrients but also acts as solid support on which the microorganisms thrive.

**Table 3:** Chemical compositions of various agro-industrial substrates used in solid-state fermentation.

Substrate composition	Substrate	Reference
Lignocellulose	Barley husk, Barley straw, Corncob, Rice husk, Rice straw, Soybean hulls, Sugar beet pulp, Sugarcane bagasse, Wheat bran, Wheat straw, Wood	Osama et al. (2013) Pensupa et al. (2013) Valentino et al. (2016) Saratale et al. (2017) Podvolotskaya et al. (2019)
Protein	Canola, Coconut, Cottonseed, Groundnut, Jatropha, Mahua cake, Mustard, Oil cakes from peanut, Olive copra, Palm kernel, Pumpkin, Rapeseed meal, Sesame, Soybean, Sunflower	Rashid et al. (2011) Shi et al. (2015) Sadh et al., 2017) Vahidi et al. (2017) Gupta et al. (2018)
Soluble sugar	Apple pomace, Carob pods, Coffee pulp, Grape pomace, Jack fruit peel, Lemon peel and pulp, Kiwi pomace, Molasses, Orange peel and pulp, Papaya peels, Peach pomace, Pineapple waste, Sugar beet pulp, Sweet sorghum stalk	Yalemtesfa et al. (2010) Hongzhang et al. (2011) Olorunnisola et al. (2017) Aruna et al. (2018)
Starch	Banana peel, Barley, Cassava meal, Cassava pulp, Cornmeal, Oats, Okara, Rice, Rice bran, Sweet potato residues, Wheat bran, Yam peels	Kupski et al. (2012) Yafetto (2018) Yafetto et al. (2020)

## 6 Applications of solid-state fermentation

### 6.1 Biodetoxification of agro-industrial wastes through solid-state fermentation

Nutrition is an important factor in the growth, and, therefore, the survival of livestock. One challenge that confronts livestock production is the availability of affordable, high-quality animal feed. Agro-industrial residues are used as feed for livestock, and because they are readily available at the farms and industries, at a little or no cost, their use reduces the cost of feeding farm animals and regulate their environmental impact as pollutants as a result of indiscriminate disposal (Yacout, 2016). Although they are acquired at a reduced or no cost, these agro-industrial wastes also present a major problem that needs to be circumvented; they contain anti-nutritional factors (hydrogen cyanide, caffeine, oxalate, tannins, polyphenols, saponins, etc.) that interfere with the bioavailability and digestibility of the nutrients present (Babalola and Giwa, 2012; Ogodo et al., 2019). The metabolism of these anti-nutritional factors in feeds yield products that decrease the presence of one or more important nutrients that are required for the proper growth and development of livestock (Yacout, 2016). Notwithstanding the presence of anti-nutritional factors in agro-industrial wastes, their toxic effects can be reduced by chemical treatment with polyethylene glycol or by the use of microorganisms through solid-state fermentation (Pandey et al., 2000a; Yacout, 2016). Solid-state fermentation, thus, presents itself as the most effective and preferred approach used in the biodetoxification of agro-industrial wastes (Joshi et al., 2014). Through solid-state fermentation, microorganisms secret a plethora of enzymes that potentially (i) improve bioavailability, digestibility, and uptake of proteins and carbohydrates through degradation and removal of anti-nutritional factors such as alkaloid, flavonoid, oxalate, phytate, and tannin from the substrates, (ii) increase the concentrations of vitamins, minerals, proteins, and amino acids, and (ii) enhance organoleptic properties (flavour, texture, appearance, and palatability) of foods (Babalola and Giwa, 2012; Ogodo et al., 2019). Oboh (2006) showed a significant decrease in cyanide and phytate content in cassava peels after 7 days of fermentation. The reduction in the cyanide was attributed to the ability of the mixed culture of *Saccharomyces cerevisiae*, *Lactobacillus delbrueckii* and *L*. *coryniformis* to partially degrade cyanogenic glucosides, whereas the decrease in the phytate content of the fermented cassava peels was attributed to the possible secretion of phytase that hydrolyses phytate, thereby reducing its content. Similarly, the content of the toxin, ricin, in castor bean cake was reduced under solid-state fermentation using *Penicillium simplicissimum* and *Paecilomyces variotii* (Godoy et al., 2009; Madeira et al., 2011). Brand et al. (2000) and Roussos et al. (1994) recorded reductions in caffeine of coffee husk and pulp using solid-state fermentation. Orozco et al. (2008) reported approximately one-third (30%) reduction of polyphenols in coffee pulp residues using solid-state fermentation. Other studies involved with the degradation of anti-nutritional, anti-physiological and toxic compounds in agro-industrial wastes using microbial fermentation include the following:

(i) gossypol in cottonseed meal by *Candida tropicalis*, *Saccharomyces cerevisiae*, and *Aspergillus niger* (Zhang et al., 2006; Khalaf and Meleigy, 2008),
(ii) phytic acid in rapeseed meal by *Aspergillus niger* (El-Batal and Karem, 2001), and canola meal by *Aspergillus carbonarius* (Al-Asheh and Duvnjak, 1994),
(iii) phorbol esters in *Jatropha* seed cake by the bacterium *Pseudomonas aeruginosa* (Joshi et al., 2011),
(iv) ochratoxin A and B in contaminated barley by *Pleurotus ostreatus* (Engelhardt, 2002),
and,
(v) β-N-oxalyl-L-α,β-diaminopropionic acid in grass pea by *Aspergillus oryzae* and *Rhizopus oligosporus* (Yigzaw et al., 2001).

From the aforementioned studies, it is evident that microbial fermentation is key to reducing the potential anti-nutritional factors of agro-industrial wastes, thereby improving the bioavailability, digestibility and uptake of proteins and carbohydrates.

### 6.2 Enzymes

Enzymes are highly efficient biocatalysts employed in many industrial processes because they have (i) unique specificity to substrates, (ii) the ability to speed up reactions that would otherwise be slow to complete, and (iii) are toxic-free (Chapman et al., 2018). Microbial production of enzymes is one of the most successful applications of solid-state fermentation. The last decade has seen extensive research into microbial enzyme production through solid-state fermentation, which has increased the number of enzymes that are produced in large quantities for commercial and industrial purposes (Couto, 2008; Thomas et al., 2013; Lizardi-Jimenez and Hernandez-Martınez, 2017; Yazid et al., 2017). Some examples of enzymes obtained from solid-state fermentation include the following: (i) cellulases (Hu et al., 2012); (ii) α-amylases (Alemu, 2013; Hashemi et al., 2013; Sharma and Satyanarayana, 2012); (iii) proteases (Malik and Shinde, 2016); (iv) lipases (Oliveira et al., 2016); (v) phytases (Bogar et al., 2003); (vi) laccases (Rosales et al., 2007); (vii) xylanases (Thomas et al., 2013); (viii) xylosidases (Diaz-Malvaez et al., 2013); (ix) chitin deacetylase (Suresh et al., 2011); and (x) invertase (Alegre et al., 2009). The production of enzymes from solid-state fermentation has been reviewed in recent years (Couto, 2008; Thomas et al., 2013; Lizardi-Jimenez and Hernandez-Martınez, 2017; Yazid et al., 2017). Several studies have amply demonstrated the use of different lignocellulosic agro-industrial wastes for the production of enzymes (Table 4). Although several industrially-produced enzymes by solid-state fermentation are reported in the literature, efforts continue to explore the possibilities of large-scale enzyme production at a more affordable cost using new sources and strains of microorganisms and available agro-industrial wastes (Thomas et al., 2013). Notable industrial applications of enzymes are in the food, beverage, detergent, biofuel, cosmetic, fabric, and pharmaceutical industries (Yazid et al., 2017; Mejias et al., 2018).

**Table 4:** Enzymes produced from agro-industrial wastes with solid-state fermentation using microbial biotechnology.

Enzyme	Microorganism	Substrate	Reference
α-amylase	*Aspergillus oryzae* *Aspergillus niger* *Penicillium chrysogenum*	Black gram bran, Corncob leaf, Coconut oil cake, Flour mill waste, Gingelly oil cake, Groundnut oil cake, Rice bran, Rye straw, Soyabean husk and meal, Tuna fish powder waste, Wheat bran, Wheat gluten waste, Wheat straw	Balkan and Ertan (2007) Kumar et al. (2011) Sahnoun et al. (2015) Melnichuk et al. (2020)
α-Galactosidase	*Aspergillus niger*	Rice bran, Rice husk, Rice polishing, Wheat bran	Awan et al. (2009)
β-fructofuranosidase	*Aspergillus tamarii*	Corncobs, Lemon peels, Oat bran, orange, Soybean, Wheat bran	de Oliveira et al. (2020)
Cellulase	*Aspergillus fumigatus* *Penicillium citrinum* *Trichoderma koningii* *Trichoderma reesei*	Oil palm trunk, Vinegar waste, Wheat bran	Liu and Yang (2007) Ang et al. (2013) Lodha et al. (2020)
Glucoamylase	*Aspergillus awamori* *Aspergillus* sp. *Fusarium solani*	Black gram bran, Green gram bran, Maize bran, Rice flakes, Rice bran, Wheat bran	Bhatti et al. (2007) Anto et al. (2006) Negi et al. (2011)
Inulinase	*Penicillium oxalicum*	Carrot pomace	Singh et al. (2018)
Lipase	*Aspergillus flavus* *Aspergillus niger* *Penicillium chrysogenum Trichoderma harzianum*	Jatropha seed cake, Rice bran, Wheat bran,	Falony et al. (2006) Toscano et al. (2013) Putri et al. (2020)
Pectinase	*Moniliella* *Penicillium* sp. *Penicillium viridicatum*	Orange bagasse, Banana peels, Corn tegument, Mango, Orange bagasse, Sugar bagasse, Wheat bran	Silva et al. (2002) Martin et al. (2004)
Pectin esterase	*Aspergillus niger*	Apple pomace	Joshi et al. (2006)
Protease	*Aspergillus awamori*	Wheat bran	Negi et al. (2011)
α-amylase, β-amylase	*Aeromonas caviae* *Anoxybacillus amylolyticus* *Bacillus subtilis*	Banana waste, Cassava bagasse, Cassava, Coconut cake, Corn bran, Cornflour, Potato peel, Rice bran, Rice husk, Sugarcane bagasse, Tea waste, Wheat bran, Wheat Straw	Mussatto et al. (2012) Finore et al. (2014) Pranay et al. (2019)
Cellulases	*Bacillus subtilis* *Streptomyces viridochromogenes*	Banana fruit stalk, Banana fruit stalk, Coconut pith Leached beet pulp, Rice husks, Rice straw, Sweet sorghum silage, Wheat bran,	El-Naggar et al. (2011) Mussatto et al. (2012)
Fibrinolytic enzyme	*Bacillus cereus* *Bacillus halodurans*	Banana peel, Black gram husk, Cow dung, Cuttlefish waste, Paddy straw, Rice bran, Wheat bran	Biji et al. (2016) Vijayaraghavan et al. (2016)
Laccase	*Rheinheimera sp.*	Peels of citrus fruits	Sharma et al. (2017)
Pectinase	*Bacillus cereus*	Orange bagasse, Rice bran, Sugarcane bagasse, Wheat bran	Namasivayam et al. (2011)

### 6.3 Single-cell proteins

The consumption of microorganisms is widely known as much as the use of mushrooms for food and food flavourings. In Germany, for example, during World War II (1939–1945), the diets of undernourished citizens were supplemented with yeasts and moulds as sources of protein (Ukaegbu-Obi, 2016). The world's population, particularly of the African continent, is continually growing, and there is the need to consider microbes as a significant source of protein, fat, and vitamins for humans as necessitated by an increase in both animal and human food supply and demand. The high demand for protein-rich foods has, therefore, led to the search for alternative protein sources to supplement the conventional animal and plant protein sources. To this end, single-cell proteins (SCP) emerged as one of the innovative approaches that sought to solve the global food problem (Ukaegbu-Obi, 2016). Research on SCP technology began a century ago when Max Delbruck and his colleagues discovered the high value of the surplus of the brewer's yeast as a feed supplement for animals (Suman et al., 2015). Since then, SCPs have become a mainstay in the production of high protein sources for animal feed and food rations for humans. The development of SCP technology is even more profound especially when many higher plant foods contain sufficient protein to supply the needs of human beings, but cannot serve as sole sources of dietary protein since their proteins are deficient in certain specific amino acids. For instance, wheat protein is low in lysine, rice protein in lysine and threonine, corn protein in tryptophan and lysine, and, bean and pea protein in methionine. Therefore, the production of SCPs for the enrichment of agro-industrial wastes through solid-state fermentation has become an important, innovative means to augment the protein deficit associated with plants (Table 5).

**Table 5:** Protein production from agro-industrial wastes with solid-state fermentation using microbial biotechnology.

Substrate	Microorganism	Reference
Apple	*Trichoderma reesei*	Abosiada et al. (2017)
Banana	*Rhizopus oryzae* *Saccharomyces cerevisiae* *Trichoderma reesei*	Abosiada et al. (2017) Fatmawati et al. (2018)
Brewery spent grain	*Rhizopus oligosporus*	Canedo et al. (2016)
Cactus pear	*Aspergillus niger* *Rhizopus* sp.	Carvalho et al. (2015)
Cassava (Peels and pulp)	*Aspergillus niger* *Aspergillus tamarii* *Saccharomyces cerevisiae* *Trichoderma viride*	Oboh (2006) Andriani et al. (2015) Yafetto (2018) Yafetto et al. (2019)
Cocoyam	*Aspergillus oryzae*	Duru and Uma (2003)
Grape marc	*Aspergillus oryzae* *Trichoderma reesei*	Zepf and Jin (2013)
Irish potato	*Aspergillus niger* *Saccharomyces cerevisiae*	Akintomide and Antai (2012)
Mango	*Trichoderma reesei*	Abosiada et al. (2017)
Olive cake	*Lentinus edodus*	Vahidi et al. (2017)
Orange	*Aspergillus niger* *Chaetomium* sp. *Trichoderma reesei*	Yalemtesfa et al. (2010) Alemu (2013) Abosiada et al. (2017)
Pineapple	*Aspergillus niger* *Saccharomyces cerevisiae* *Trichoderma viride*	Adu et al. (2018) Aruna et al. (2018)
Rapeseed cake	*Aspergillus niger*	Shi et al. (2015)
Rice bran	*Rhizopus oryzae*	Kupski et al. (2012)
Rice straw	*Aspergillus flavus* *Aspergillus niger* *Aspergillus ochraceus* *Penicillium citrinum*	Valentino et al. (2016)
Sorghum stalk	*Candida tropicalis*	Hongzhang et al. (2011)
Sugarcane bagasse	*Cladosporium cladosporioides* *Fusarium semitectum* *Monascus ruber*	Valentino et al. (2016)
Sweet potato	*Aspergillus niger*	Adu et al. (2018)
Tomato	*Trichoderma reesei*	Abosiada et al. (2017)
Watermelon	*Aspergillus niger*	Adu et al. (2018)
Yam	*Aspergillus niger* *Saccharomyces cerevisiae* *Trichoderma viride*	Aruna et al. (2018) Yafetto et al. (2020)

### 6.4 Organic acids

Generally, organic acids are produced by biological and chemical synthesis (Yazid et al., 2017). However, because chemically-synthesized substances can have residual, deleterious effects on humans, researchers have explored the potential for biological systems as alternatives for biologically-produced substances, including organic acids. One alternative to the chemical synthesis of organic acids is microbial fermentation. Biologically-produced organic acids are deemed safe, cost-effective and easy to produce, and they are the third-largest produced organic products after enzymes and secondary metabolites (Ali and Zulkali, 2011). Like enzymes, organic acids are applied in food, beverage, medical, pharmaceutical, and cosmetic industries, among others. The food industry uses the largest amount of organic acid products followed by the medical and pharmaceutical industries. Presently, a large number of organic acids is produced by solid-state fermentation, a technology that has emerged as a cheaper option to submerged fermentation (Lizardi-Jimenez and Hernandez-Martınez, 2017). Some organic acids produced by solid-state fermentation using different agro-industrial wastes include butyric acid, citric acid, ellagic acid, fumaric acid, gallic acid, gluconic acid, lactic acid, oxalic acid, succinic acid, among others (Table 6).

**Table 6:** Organic acids produced from agro-industrial wastes with solid-state fermentation using microbial biotechnology.

Organic acid	Microorganism	Substrate	Reference
Acetic acid	*Lactobacillus casei* *Lactobacillus delbrueckii*	Papaya peels, Pineapple peels	Raji et al. (2012) Vikas and Mridul (2014)
Butyric Acid	*Escherichia coli* *Lactobacillus plantarum*	Pineapple peels, Rice bran, Wheat bran	Sjöblom et al. (2015) Akhtar et al. (2020)
Chlorogenic acid	*Aspergillus niger*	Coffee pulp	da Silveira et al. (2019)
Citric acid	*Aspergillus niger* *Aspergillus oryzae* *Gibberella fujikuroi*	Banana peel, Grapes, Mosambi peel and bagasse, Oat bran, Orange peel, Pineapple peel, Semi-dried fig, Sugarcane bagasse and molasses, Sweet lime peel, Wheat bran, Wheat straw	Roukas (2000) Kumar et al. (2002) Al-Mahin et al. (2008) Khosravi Darani et al. (2008) Goud et al. (2012) Rao and Reddy (2013) Hussain (2019) Subramaniyan et al. (2019) Bastos and Ribeiro (2020)
Fumaric acid	*Aspergillus niger*	Soybean cake, Sugar, Molasses	Papadaki et al. (2018)
Gallic Acid	*Aspergillus niger* *Trichoderma reesei*	Apple peels, Apple seeds, Banana peels, Black plum seeds, Guava seeds, Mango peels and seeds, Pomegranate peels, Tamarind seeds, Watermelon seeds	Arshad et al. (2019) Saeed et al. (2020)
Gibberellic acid	*Saccharomyces cerevisiae*	Crude rice bran, Malt residue	Werle et al. (2020)
Gluconic acid	*Gibberella fujikuroi*	Semi-dried fig	Roukas (2000)
Humic acid	*Actinobacillus succinogenes*	Fruit bunch fibres, Oil palm	Volpi et al. (2019)
Lactic acid	*Actinobacillus succinogenes* *Aspergillus oryzae* *Bacillus subtilis* *Lactobacillus delbruecki* *Lactobacillus spp.*	Cassava bagasse, Cassava fibrous residue, Cassava residues, Green peas, Mango peels, Orange, Potato peels, Red lentil flour, Sweetcorn, Whey	Hofvendah and Hahn-Hagerdal (2000) Altaf et al. (2007) John et al. (2007) Ray et al. (2008) Mudaliyar et al. (2012) Jawad et al. (2013)
Poly-ɣ-glutamic acid	*Bacillus subtilis*	Swine manure	Chen (2005)
Succinic acid	*Aspergillus niger* *Lactobacillus amylophilus* *Rhizopus arrhizus*	Banana, Cull peaches, Onion, Orange, Pineapple, Potato, Sugarcane molasses, Tomato, Watermelon, Wheat	Du et al. (2007) Krishnakumar (2013) Dessie et al. (2018)

Citric acid and lactic acid are the most widely used commercial and industrial organic acids. *A*. *niger* is the most widely reported fungus used in the production of citric acid through solid-state fermentation (Table 6; Yazid et al., 2017). Agro-industrial wastes used for the production of citric acid are those obtained from fruits such as apple pomace (Dhillon et al., 2011a), grape peel (Goud et al., 2012), orange peel (Hamdy, 2013), banana peel (Karthikeyan and Sivakumar, 2010), pineapple pulp waste (Bezalwar et al., 2013), and mixed fruit waste (Kumar et al., 2003). Other agro-industrial wastes like sugarcane bagasse, coffee husk, empty fruit bunches of oil palm, cassava bagasse, peanut shell, corn grits, pulp and paper solid wastes are also utilized to produce citric acid under conditions of solid-state fermentation (Pandey et al., 2000b,c,d; Soccol et al., 2017; Yazid et al., 2017). Torrado et al. (2011) compared citric acid yield under solid-state fermentation to submerged fermentation using orange peels with *Aspergillus niger* and reported that the production of citric acid in solid-state fermentation was three times more than that of submerged fermentation.

Lactic acid, a popular preservative and acidifying agent used in the food industry is another important organic acid produced through solid-state fermentation. Due to high demand, agro-industrial wastes have been employed as the substrates to produce lactic acid on a large scale under solid-state fermentation compared to submerged fermentation. Lactic acid bacteria such as *Lactobacillus amylophilus* (Altaf et al., 2006), *L. delbrueckii* (John et al., 2006)*, L. casei* (Qi and Yao, 2007)*,* and *L. plantarum* (Gowdhaman et al., 2012) are the most commonly used bacteria employed in lactic acid production (Table 6).

### 6.5 Biofuel

Biofuels are renewable energy resources produced from bio-based raw materials. They are important because of their appropriateness as a replacement for petroleum fuels that are obtained from crude oil. Bioethanol and biogas are the most preferred among the nonconventional energy resources (Panda and Ray, 2015). Bioethanol, for example, is adopted in Brazil, China and the United States of America, and it is currently the most extensively used biological fuel in the world. Its production has decreased the consumption of crude oil that is obtained from fossil fuels, consequently, reducing carbon dioxide emissions and environmental pollution. Several kinds of agro-industrial wastes have been used to produce bioethanol through solid-state fermentation (Table 7). The production of ethanol is traditionally accomplished by submerged fermentation, but studies suggest solid-state fermentation as a more feasible approach because it utilizes agro-industrial wastes both as solid support and a carbon source. Solid-state fermentation further presents itself as the better option for ethanol production because of its lower water requirement, smaller volumes of fermentation mash, and disposal of less liquid water, hence less impact of environmental pollution (Bhargav et al., 2008; Lizardi-Jimenez and Hernandez-Martınez, 2017). Different species of filamentous fungi and yeasts have been reported for their ability to produce ethanol by solid-state fermentation. Examples of these fungi are *A. niger, Aspergillus variabilis, Fusarium oxysporum, Penicillium* sp. *Trichoderma* sp*.* as well as *Candida pulcherrima, Candida stellata, Hansenula anomala, Kloeckera apiculata*, and *Saccharomyces cerevisiae* (Bhargav et al., 2008; Yazid et al., 2017). Several studies show that *Saccharomyces cerevisiae* is widely used for the bioconversion of solid wastes such as apple pomace (Kanwar et al., 2012), grape and sugar beet pomace (Rodríguez et al., 2010), potato peel (Chintagunta et al., 2016), sweet sorghum stalks (Du et al., 2014), sugarcane bagasse (Liu et al., 2015b), mixed food waste (Kiran and Liu, 2015), etc., into bioethanol under conditions of solid-state fermentation. Like other bioproducts obtained from solid-state fermentation through the utilization of fungi, bacteria are also equally employed for ethanol production through the bioconversion of agro-industrial wastes (Table 7). For instance, high bioethanol yield was recorded from the bioconversion of sweet sorghum bagasse by *Zymomonas mobilis* (Yu et al., 2008, 2014, 2016)*,* and switchgrass by *Clostridium phytofermentans* (Jain et al., 2013) using solid-state fermentation. The mechanisms involved in the utilization of components of agro-industrial residue such as hemicellulose, lignocellulose, and other carbohydrates to synthesize simple sugars for subsequent conversion into bioethanol, biodiesel, biogas, and the LCA and TEA of their production is beyond the scope of this review.

**Table 7:** Most frequently explored topics in the field of solid-state fermentation.

S/N	Core Research Topic	Number of publications	Per cent in a total publication
1	Enzymes	2354	60.87
2	Antioxidants	303	7.83
3	Animal feed	214	5.53
4	Biofuel	162	4.20
5	Agricultural wastes	130	3.36
6	Bioethanol	113	2.92
7	Secondary metabolites	106	2.74
8	Antibiotics	94	2.43
9	Organic acids	86	2.22
10	Biodiesel	81	2.10
11	Protein enrichment	70	1.81
12	Biosurfactants	47	1.21
13	Biogas	36	0.93
14	Biopesticides	33	0.85
15	Active compounds	22	0.57
16	Biofertilizers	16	0.41
**Total**		**3867**	**100**

### 6.6 Biofertilizer

Agricultural wastes are composted to produce manure for use as soil enhancers particularly in developing countries, where advanced, mechanized farming is not practised. At best, governments supply subsidised chemical fertilisers to farmers to apply to soils during the planting season to increase crop yield. But the continuous use of these chemical fertilizers harms the soil. Therefore, to curb this situation of applying chemical fertilizers to soils, researchers have explored the potential for biologically-produced biofertilizers that enhance plant growth, development and crop yield (Alam and Seth, 2012). Ngampimol and Kunathigan (2008) described biofertilizers as fertilizers obtained from agro-industrial wastes in which live microorganisms are present, and where their activities enhance the nutrient quality of the agro-industrial wastes. Nutrients in the biofertilizers enhance the soil's nutritive qualities and are of benefit to the plants. Production of large quantities of agricultural produce, as a result of the high demand for food to feed the increasing global population, yields a huge amount of agro-industrial wastes (Diacono et al., 2019). These agro-industrial wastes could be converted into biofertilizers to augment agricultural lands to sustain the production of large quantities of agricultural produce. Recent studies have focused on biologically converting agricultural wastes into biofertilizers using solid-state fermentation techniques. Lim and Matu (2015) utilized wastes from banana, watermelon, pineapple, papaya, and citrus orange to produce biofertilizer using solid-state fermentation, where the final product was then applied in the cultivation of vegetables. Similarly, Alam and Seth (2012) compared chemical fertilizers and biofertilizers by investigating their effect on the growth, development, and yield of rice (*Oryza sativa*). The result showed that rice plants treated with biofertilizers produced the highest plants height with the highest yield of rice in contrast to rice plants treated with chemical fertilizer, which produced the lowest plant heights with the lowest yields. Spent mushroom compost from *Pleurotus eous* used as biofertilizer to augment soil supported better growth in tomato (*Solanum lycopersicon*) and pepper (*Capsicum annuum* L.) under greenhouse conditions (Wiafe-Kwagyan and Odamtten, 2018). Findings from these studies suggest that solid-state fermentation, through the application of microbial biotechnology, can be used to produce biofertilizers, which has the potential to change the face of agriculture globally.

## 7 Bibliometric analysis of research on solid-state fermentation

The term *bibliometrics* was first coined by Alan Pritchard in 1969 to refer to *the application of mathematics and statistical methods* to the analysis of scientific publications. Even before the term was introduced, Cole and Eales (1917) and Wilson and Fred (1935) had conducted quantitative analyses of publication information of the scientific literature. For example, Cole and Eales (1917) conducted a statistical analysis of more than three centuries of publication in comparative anatomy, during which they assessed the evolution of research in the field and the amount of contribution each European country has made toward its growth (Lei and Liu, 2019). The field of Bibliometrics has since evolved, and its importance has soared among academics, researchers and policymakers. Presently, the importance of bibliometry is paramount to professional academics because they need to know (i) what research topics are most popular (and arguably the most important) and (ii) which publications (journal articles, books, and book chapters) and authors are most influential in their disciplines. This has helped academic professionals to stay current regarding research trends in their respective fields and to make informed decisions about what research issues to investigate. Additionally, information obtained from bibliometric analyses of the literature has helped academic institutions and policymakers of government and private agencies to make more informed decisions about the allocation of research funding (Lei and Liu, 2019). Thus, bibliometric analysis is a useful statistical approach that can be used to quantitatively analyze the current state of scientific research, by highlighting trends in the field as well as gaps in the literature (Kim et al., 2016; Fawzy et al., 2020). This section of the paper, therefore, focused on the bibliometric analysis of research trends in bioconversion of agro-industrial wastes by microbial biotechnology with solid-state fermentation from 1970 to 2020 specifically to answer the following questions:

(i) what are the major types of publications?
(ii) what are the most frequently explored topics?
(iii) what are the most highly cited publications and authors?
(iv) what are the most highly cited journals? and
(v) which country/region has the most cited publications?

### 7.1 Types of publication, publication trends, and core research topics

**Figure 2:**
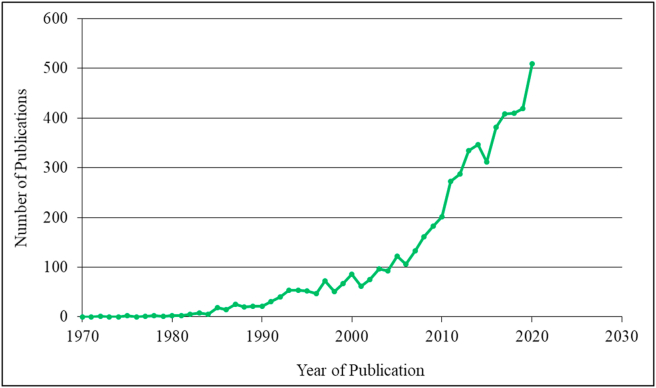
Publications of research findings in solid-state fermentation from 1970 to 2020.

Based on literature search and analysis from Dimensions database, a total of 5630 publications (5208 articles (92.50%); 340 book chapters (6.04%); 39 preprints (0.69%); 32 proceedings (0.56%); 8 edited books (0.14%); 3 monographs (0.05) specifically on solid-state fermentation were retrieved. It is instructive to note that these 5208 articles included reviews that had been published in the field. Thus, research articles and reviews were the most common types of publications used to disseminate findings of studies in solid-state fermentation. This present search surprisingly revealed that no bibliometric study has been conducted nor published on solid-state fermentation as no record of such study was found in any of the databases (Dimensions, Scopus, Web of Science, ScienceDirect, and PubMed). This provided an impetus to pursue the present bibliometric analysis of the literature on solid-state fermentation. To this end, data were analyzed by comparing the number of publications per year over the period under review. Interestingly, the data show that there has been a steady, consistent increase in the number of publications from 1970 until 2000, after which there was an astronomic increase till 2020 (Figure 2). This increasing trend suggests a correlation between increased interest in research and a corresponding increase in publication outputs and outlets for the dissemination of research findings in the field. The increase in publications may also be attributed to improvements in methods and techniques that have enhanced state-of-the-art laboratory and field research, where findings could be implemented in bioreactors on large, industrial scales.

Based on data from literature search and analysis, a total of 16 core research topics that relate to products obtained from solid-state fermentation were identified (Table 7). “Enzymes” was the most dominant research term during the last 50 years (2354 publications or 60.87%), followed by “antioxidants” (303 publications or 7.83%), “animal feed” (214 publications or 5.53%), “biofuel” (162 publications or 4.20%), and “agricultural wastes” (130 publications or 3.60%) (Table 7). “Agricultural wastes”, was the exception on the list of core research topics because they are not products from solid-state fermentation, but rather they constitute substrates used for the fermentation process from which products are obtained. Interestingly, “biofertilizers” was the least researched topic with 16 publications. This has implications for the agricultural sector of any economy since agro-industrial wastes have huge potential for the production of biofertilizers through microbial biotechnology and solid-state fermentation as a safer, inexpensive alternative to chemical fertilizers to improve agricultural yields of crops.

Surprisingly, more than 2000 publications on the production of enzymes through solid-state fermentation were obtained from the search. This finding is key because it accentuates the continued efforts that are made to explore the possibilities of large-scale enzyme production using new sources and strains of microorganisms and available agro-industrial wastes (Table 4; Thomas et al., 2013). It is also important to highlight that enzymes are particularly applied in the food and beverage, detergent, biofuel, cosmetic, fabric, and pharmaceutical industries, where the food industry accounts for 45% of the overall use of enzymes, followed by the detergent industry (35%), textiles industry (10%) and leather industry (3%) (Yazid et al., 2017; Mejias et al., 2018). The present data also illuminate the recent surge in interest in studies that explore the application of solid-state fermentation for the production of biosurfactants (Colla et al., 2010; Kiran et al., 2010; Makkar et al., 2011; Sobrinho et al., 2013; Bhardwaj et al., 2015; Velioğlu and Ürek, 2015; Reis et al., 2018; Sanches et al., 2018). The number of core research topics extensively explored for the production of a plethora of useful products (Table 7) further emphasizes the increasing interest in the industrial application of microbes in the valorization of agricultural wastes by solid-state fermentation.

### 7.2 Most highly cited publications, authors, and countries with the highest numbers of publications

Two lists were generated from data analysed to identify the most highly cited publications from literature retrieved from Dimensions: one list considered all the cited publications from 2010 to 2020 (Table 8); the other list focused on cited publications that were published between 1970 and 2020 (Table 9). The decision to consider publications between 2010 and 2020 is based on the argument that, when all factors are held constant, a publication that is between 1 and 3 years old tends to generate fewer citations than publications that are 5 years or older (Lei and Liu, 2019). This age effect on citation numbers is evidenced in Table 8, where only two highly cited publications, Sadh et al.’s (2018) *Agro-industrial wastes and their utilization using solid state fermentation: a review* (C#207) and Soccol et al.’s (2017) *Recent developments and innovations in solid state fermentation* (C#168), were respectively 3 and 4 years old at the time the literature search for this study was conducted on the 17^th^ March 2021. Thus, an overwhelming 18 highly cited publications were more than 4 years (Table 8). This trend was further evidenced when the most highly cited publications from 1970 to 2020 were analysed (Table 9), where de Souza and Magalhães’ (2010) *Application of microbial α-amylase in industry - A review* (C#382), and Martins et al.’s (2011) *Bioactive phenolic compounds: Production and extraction by solid-state fermentation: A review* (C#368) ranked 9^th^ and 10^th^ in the 1970–2020 category, unlike in Table 8 where they ranked 1^st^ and 2^nd^ in the 2010–2020 category. This was because most publications in Table 9 were much older than those in Table 8. Further analysis showed that the top 6 most highly cited publications were older than de Souza and Magalhães’ (2010) and Martins et al.’s (2011) publications (Table 9). This trend confirms Lei and Liu's (2019) assertion earlier expounded and demonstrates that more recent publications from studies in solid-state fermentation are likely to generate fewer citations compared to older publications. Interestingly, the oldest publication in the top 20 most highly cited publications list in Table 9 was Lonsane et al.’s (1985) *Engineering aspects of solid state fermentation* ranked 13^th^ with 353 citations. Another observation was that majority of the most highly cited publications in both categories were review articles, i.e., 12 review articles were among the top 20 publications between 2010 and 2020, whereas 18 review articles were among the top 20 publications between 1970 and 2020 (Tables 8 and 9). Interestingly, the most highly cited publication since 1970 is Pandey et al.’s (2000b) *Biotechnological potential of agro-industrial residues. I: sugarcane bagasse* with 735 citations. Dimension's database summary of Pandey et al.’s (2000b) publication states that: “*This publication in Bioresource Technology has been cited 735 times. 21% of its citations have been received in the past two years, which is higher than you might expect, suggesting that it is currently receiving a lot of interest. Compared to other publications in the same field, this publication is extremely highly cited and has received approximately 60 times more citations than average*.” Ashok Pandey also has 6 publications on the list of the most highly cited publications in Table 9. None of the book chapters, preprints, proceedings, edited books and monographs retrieved from the literature search was among the top 20 most cited publications in the two lists generated from the analyses.

**Table 8:** Top 20 most highly cited publications between 2010 and 2020.^a^

1. Application of microbial α-amylase in the industry - A review. de Souza and Magalhães (2010)*Brazilian Journal of Microbiology* C#382
2. Bioactive phenolic compounds: Production and extraction by solid-state fermentation. A review. Martins et al. (2011)*Biotechnology Advances* C#368
3. Current developments in solid-state fermentation. Thomas et al. (2013) Biochemical Engineering Journal C#274
4. Overview of Fungal Lipase: A Review. Singh and Mukhopadhyay (2012)*Applied Biochemistry and Biotechnology* C#228
5. Poly (glutamic acid) – An emerging biopolymer of commercial interest. Bajaj and Singhal (2011)*Bioresource Technology* C#225
6. Agro-industrial wastes and their utilization using solid state fermentation: a review. Sadh et al. (2018)*Bioresources and Bioprocessing* C#207
7. Production of a cellulolytic enzyme system in mixed-culture solid-state fermentation of soybean hulls supplemented with wheat bran. Brijwani et al. (2010)*Process Biochemistry* C#204
8. Fungal pretreatment: An alternative in second-generation ethanol from wheat straw. Salvachúa et al. (2011)*Bioresource Technology* C#202
9. Optimization of cellulase production by a brown rot fungus *Fomitopsis* sp. RCK2010 under solid state fermentation. Deswal et al. (2011)*Bioresource Technology* C#180
10. A pyrosequencing-based metagenomic study of methane-producing microbial community in solid-state biogas reactor. Li et al. (2013)*Biotechnology for Biofuels* C#179
11. Production of cellulases from *Aspergillus niger* NS-2 in solid state fermentation on agricultural and kitchen waste residues. Bansal et al. (2012)*Waste Management* C#169
12. Recent developments and innovations in solid state fermentation. Soccol et al. (2017)*Biotechnology Research and Innovation* C#168
13. Utilisation of waste bread for fermentative succinic acid production. Leung et al. (2012)*Biochemical Engineering Journal* C#144
14. Lipids from heterotrophic microbes: advances in metabolism research. Kosa and Ragauskas (2011)*Trends in Biotechnology* C#141
15. Solid-state fermentation: Physiology of solid medium, its molecular basis and applications. Barrios-González (2012)*Process Biochemistry* C#136
16. Recent advances in citric acid bio-production and recovery. Dhillon et al. (2011b)*Food and Bioprocess Technology* C#133
17. Value-addition of agricultural wastes for augmented cellulase and xylanase production through solid-state tray fermentation employing mixed-culture of fungi. Dhillon et al. (2011c)*Industrial Crops and Products* C#131
18. Production of cellulases and xylanase by *Aspergillus fumigatus* SK1 using untreated oil palm trunk through solid state fermentation. Ang et al. (2013)*Process Biochemistry* C#128
19. A biotechnology perspective of fungal proteases. de Souza et al. (2015)*Brazilian Journal of Microbiology* C#123
20. Solid state fermentation for production of microbial cellulases: Recent advances and improvement strategies. Behera and Ray (2016)*International Journal of Biological Macromolecules* C#120

a To save space, no full citation information is given: only the title of the article, author name, and year of publication are given (in parentheses) with the number of citations presented as C#.

**Table 9:** Top 20 most highly cited publications between 1970 and 2020.^a^

1. Biotechnological potential of agro-industrial residues. I: sugarcane bagasse. Pandey et al. (2000b)*Bioresource Technology* C#735
2. Solid-state fermentation. Pandey (2003)*Biochemical Engineering Journal* C#717
3. New developments in solid state fermentation: I-bioprocesses and products. Pandey et al. (2000a)*Process Biochemistry* C#687
4. Recent advances in solid-state fermentation. Singhania et al. (2009)*Biochemical Engineering Journal* C#470
5. Biotechnological advantages of laboratory-scale solid-state fermentation with fungi. Hölker et al. (2004)*Applied Microbiology and Biotechnology* C#432
6. Physical removal of textile dyes from effluents and solid-state fermentation of dye-adsorbed agricultural residues. Nigam et al. (2000)*Bioresource Technology* C#431
7. Transformation of vegetable waste into value added products: (A) the upgrading concept; (B) practical implementations. Laufenberg et al. (2003)*Bioresource Technology* C#427
8. Application of solid-state fermentation to food industry - A review. Couto and Sanroman (2006)*Journal of Food Engineering* C#398
9. Application of microbial α-amylase in industry - A review. de Souza and Magalhães (2010)*Brazilian Journal of Microbiology* C#382
10. Bioactive phenolic compounds: Production and extraction by solid-state fermentation. A review. Martins et al. (2011)*Biotechnology Advances* C#368
11. Recent process developments in solid-state fermentation. Pandey (1992)*Process Biochemistry* C#365
12. Solid-State Fermentation Systems - An Overview. Krishna (2005)*Critical Reviews in Biotechnology* C#354
13. Engineering aspects of solid state fermentation. Lonsane et al. (1985)*Enzyme and Microbial Technology* C#353
14. Cellulase production using biomass feed stock and its application in lignocellulose saccharification for bio-ethanol production. Sukumaran et al. (2009)*Renewable Energy* C#327
15. A review on microbial lipases production. Treichel et al. (2010)*Food and Bioprocess Technology* C#289
16. Biotechnological potential of coffee pulp and coffee husk for bioprocesses. Pandey et al. (2000c)*Biochemical Engineering Journal* C#288
17. Value-added food: Single cell protein. Anapama and Ravindra (2000)*Biotechnology Advances* C#287
18. Bioreactors for tissue mass culture: Design, characterization, and recent advances. Martin and Vermette. (2005)*Biomaterials* C#274
18. Current developments in solid-state fermentation. Thomas et al. (2013)*Biochemical Engineering Journal* C#274
20. Biotechnological potential of agro-industrial residues. II: cassava bagasse. Pandey et al. (2000d)*Bioresource Technology* C#268

a To save space, no full citation information is given: only the title of the article, author name, and year of publication are given (in parentheses) with the number of citations presented as C#. Publications 18 and 19 are tied at their given rank with the same number of citations at C#274.

Table 10 reports, in ranking order, the top 10 most highly cited authors, their country of origin, their number of publications, their raw number of citations and the corresponding citations mean. Ashok Pandey, from India, was the most cited author (123 publications and 8,613 citations) followed by Carlos Ricardo Soccol, from Brazil, who had 109 publications and 5,033 citations. Indeed, 6 of the 10 most cited authors were from Brazil with two authors, Sevastianos Roussos and Cristόbal Nόe Aguilar, from France and Mexico, respectively. The present study suggests that scholars particularly from Third World countries may be conducting more studies in solid-state fermentation and publishing their findings in reputable international journals, unlike scholars from countries that are often regarded to have long research and publication traditions. The increasing number of authors from these Third World countries (Brazil, China, India) may be further attributed to increased government funding and research support (Zhou et al., 2009; Qiu 2010; Zhang et al., 2013). This emphasizes the crucial role that government funding and support may play in research productivity and publication outputs.

**Table 10:** Frequency rank of the most cited authors (total n = 5630 publications) from 1970 to 2020.

Rank	Author	Country	Number of Publications	Citations	Citations Mean
1	Ashok Pandey	India	123	8,613	70.02
2	Carlos Ricardo Soccol	Brazil	109	5, 033	46.17
3	David Alexander Mitchell	Brazil	60	2,397	39.95
4	Denise Maria Guimarães Freire	Brazil	75	2,122	28.29
5	B. K. Lonsane	India	44	1,827	41.52
6	Sevastianos Roussos	France	65	1,740	26.77
7	Cristόbal Nόe Aguilar	Mexico	66	1,539	23.32
8	Eleni Gomes	Brazil	43	1,215	28.26
9	Nadia Kreiger	Brazil	37	1,147	31.00
10	Marcio Antonio Mazutti	Brazil	39	734	18.82

A trend of interest is that most of the highly cited authors do not have any publication listed in the top 20 most highly cited publications (Tables 8 and 9), and the following possible scenarios may have contributed to this trend:

(i) the authors may have individual publications that have fewer citations, but collectively, these publications record a high total number of citations; and
(ii) because recent publications tend to have fewer citations than older publications, authors with most recent publications may have been cited fewer times than those with older publications (Lei and Liu, 2019).

**Figure 3:**
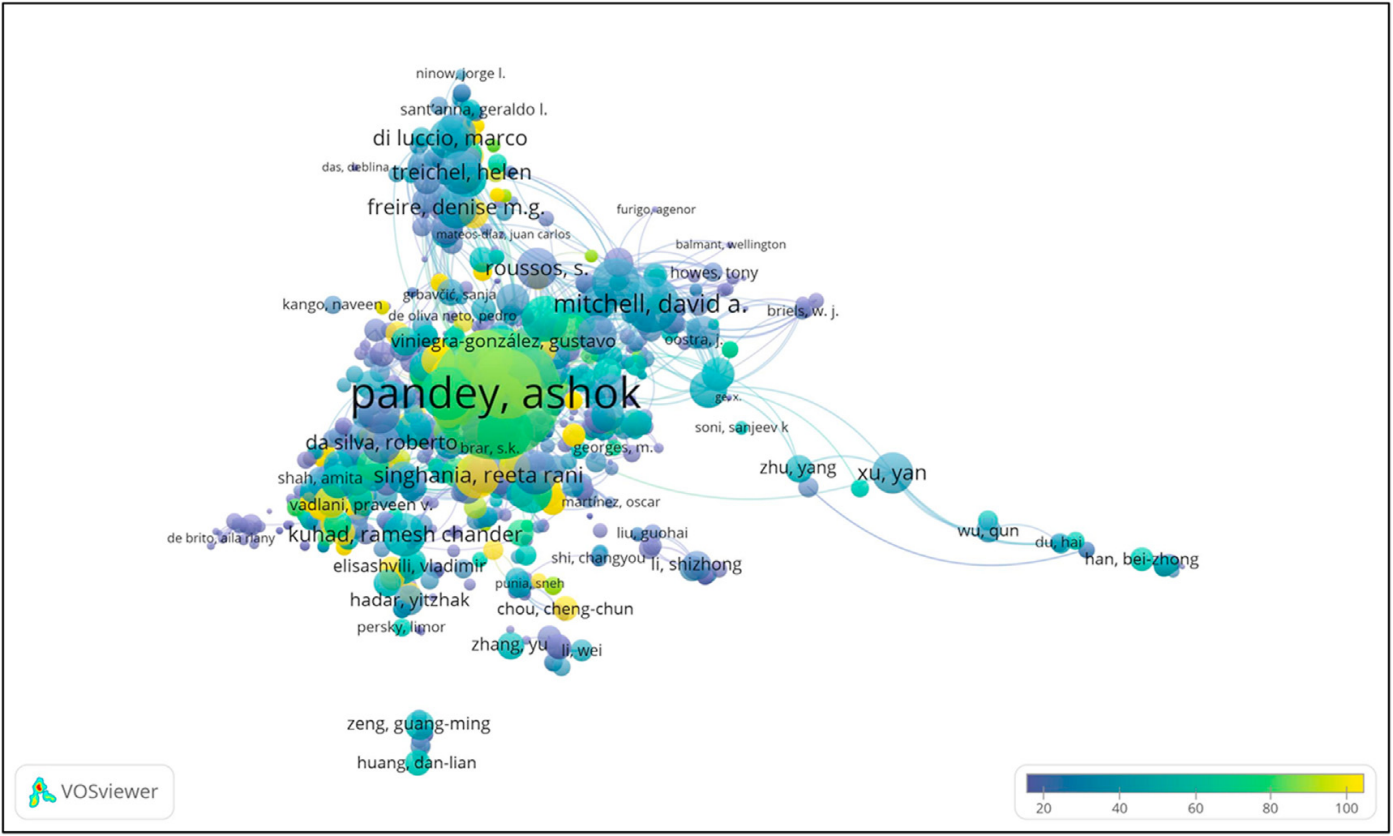
Author citation overlay visualization in studies of solid-state fermentation. Only the top 1000 publications are presented. Items with a higher weight are shown more prominently than items with a lower weight. Colour bars in the visualization map indicate the average citation per author; authors coloured yellow have a higher average citation of publications than authors coloured blue.

Bibliometric analysis of the literature with VOSviewer revealed 11 clusters of author citations (Figure 3). In the visualization of the resulting map, Ashok Pandey was located in Cluster 6 with 884 citation links and a total citation link strength of 7666, compared to Marcio Antonio Mazutti, the 10^th^ most cited author who was located in Cluster 10 with 322 citation links and a total citation link strength of 786. This analysis further confirmed that Ashok Pandey has a more compelling citation profile on the list of most highly cited authors, and he is more prominently shown on the map than Marcio Antonio Mazutti (Figure 3).

### 7.3 Highly cited journals

Table 11 reports, in ranking order, the top 10 most highly cited journals, their number of publications, their raw number of citations and the corresponding citations mean. *Bioresource Technology* was the most cited journal (12,394 citations) followed by *Process Biochemistry* (9,997 citations). Further analysis of the data revealed that some of the journals with fewer publications were highly cited than those with a higher number of publications (Table 11), a similar trend observed in Table 9. For example, although *Biochemical Engineering Journal* has 87 publications, compared to *Applied Microbiology and Biotechnology* (105 publications), the former is ranked 3^rd^ with 5,698 citations, whereas the latter is ranked 4^th^ with 4,790 citations (Table 11). The reasons adduced for the trend observed for the most cited authors in Table 9 apply to the trend observed for the most cited journals in Table 11. The top 10 journals identified from the database reveal a broad scope of interests in solid-state fermentation within notable fields such as biochemistry, engineering, microbiology, and biotechnology that suggests the inter and multidisciplinary state of the field of solid-state fermentation.

**Table 11:** Frequency rank of most cited journals (total n = 5630 publications) from 1970 to 2020.

Rank	Journal	Number of Publications	Citations	Citations Mean
1	Bioresource Technology	223	12,394	55.58
2	Process Biochemistry	190	9,797	51.56
3	Biochemical Engineering Journal	87	5,698	65.49
4	Applied Microbiology and Biotechnology	105	4,790	45.62
5	Enzyme and Microbial Technology	73	3,893	53.33
6	Applied Biochemistry and Biotechnology	175	3,626	20.72
7	World Journal of Microbiology and Biotechnology	120	2,857	23.81
8	Biotechnology Letters	89	2,031	22.82
9	Bioprocess and Biosystems Engineering	89	1,508	16.94
10	Biocatalysis and Agricultural Biotechnology	97	1,019	10.51

**Figure 4:**
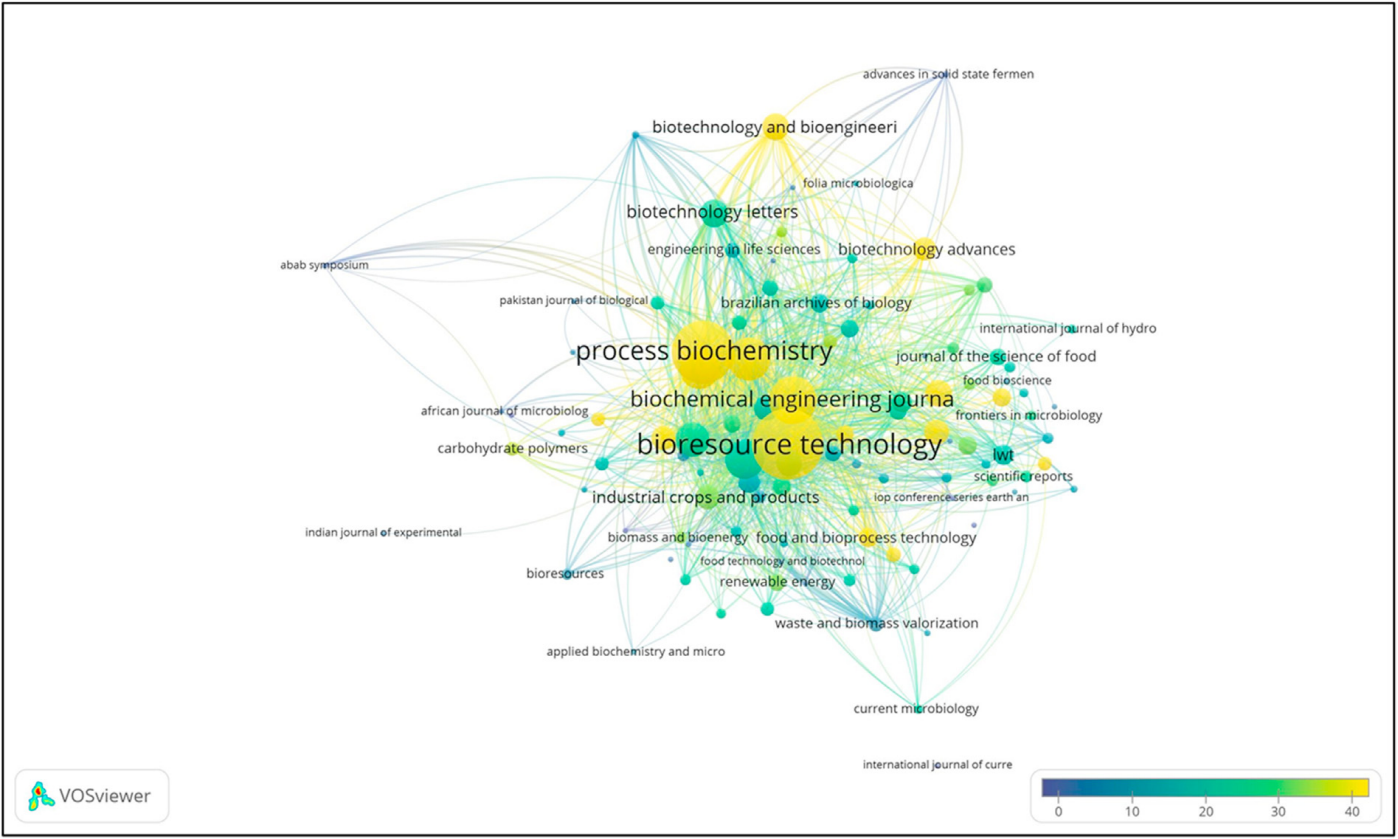
Journal citation overlay visualization in studies of solid-state fermentation. Only the top 1000 publications are presented. Items with a higher weight are shown more prominently than items with a lower weight. Colour bars in the visualization map indicate the average citation per author; authors coloured yellow have a higher average citation of publications than authors coloured blue.

Bibliometric analysis of the data with VOSviewer revealed 9 clusters of journal citations (Figure 4). In the visualization of the resulting map, *Bioresource Technology* was located in Cluster 2 with 102 citation links and a total citation link strength of 3038, compared to *Biocatalysis and Agricultural Biotechnology*, the 10^th^ most cited journal located in Cluster 6 with 89 citation links and a total citation link strength of 882. Correspondingly, *Bioresource Technology* is more prominently shown on the map than *Biocatalysis and Agricultural Biotechnology* (Figure 4).

## 8 Limitations

This paper reviewed the pertinent literature between 1970 and 2020 and provided information on the application of solid-state fermentation in the production of useful industrial products mainly from agro-industrial wastes. The paper was further strengthened by the large amount of data that supported the bibliometric analysis of the literature. However, the study may be limited by (i) the kind of published materials included in the analysis of the database (e.g., journals, conference papers, books, book chapters); (ii) the kind of published materials that fall within the subject scope of the database (subject breadth); (iii) how much of scholarly output on solid-state fermentation was included in the database (subject depth); (iv) the possible exclusion of publications on solid-state fermentation that emanate from particular geographic regions (geographic coverage); (v) the use of only English-written publications exclusive of other key languages (language coverage); and (vi) depth of backfiles (e.g., how far back are citations tracked). Moreover, data could be impacted also by the unethical behaviour of authors such as Salami publishing, self-plagiarism, honorary authorship, and self-citation.

## 9 Conclusions

This paper highlighted the importance of the application of solid-state fermentation for the bioprocessing of various agro-industrial wastes for value addition. The production of enzymes remains the most dominant research interest in the field. Research on solid-state fermentation has increased exponentially between 1970 and 2020 as a result of increasing prospects of using solid-state fermentation to valorize a wide range of agro-industrial wastes into products of enormous industrial, agricultural, and health benefits to humans. There is a steady increase also in publications from emerging economies like China, India, and Brazil that suggests an immense interest by researchers to process agro-industrial wastes into useful domestic and industrial products, while remediating the environment from unwarranted pollution Microbiologists and scientists from allied fields should further explore new areas of interest, where collaborative, interdisciplinary efforts could be enhanced. The bibliometric analysis generated large quantitative data that revealed important research trends in solid-state fermentation. This approach of analyzing various types of data was crucial since one set of data was inadequate to understand research trends with bibliometric analysis. Most of the highly cited authors did not have any publication listed in the top 20 most highly cited publications and vice versa, a finding that emphasizes the need to analyze various data sets to obtain a comprehensive, comparable trend. Although a bibliometric study is quantitative, it involves qualitative analysis that requires subjectivity in decisions that consider what variables to analyze, and what constitutes a research topic as well as research trend. More bibliometric studies on other aspects of research on solid-state fermentation are required to augment the findings of this study.

## Declarations

### Author contribution statement

All authors listed have significantly contributed to the development and the writing of this article.

### Funding statement

This research did not receive any specific grant from funding agencies in the public, commercial, or not-for-profit sectors.

### Data availability statement

Data included in article/supplementary material/referenced in article.

### Declaration of interest’s statement

The authors declare no conflict of interest.

### Additional information

No additional information is available for this paper.
